# Optimization of Cultural Conditions for Antioxidant Exopolysaccharides from* Xerocomus badius* Grown in Shrimp Byproduct

**DOI:** 10.1155/2016/2043787

**Published:** 2016-02-22

**Authors:** Xiujun Gao, Peisheng Yan, Xin Liu, Jianbing Wang, Jiajia Yu

**Affiliations:** ^1^School of Marine Science and Technology, Harbin Institute of Technology at Weihai, Weihai 264209, China; ^2^Department of Biological & Agricultural Engineering, North Carolina State University, Raleigh, NC 27695, USA

## Abstract

To optimize the production conditions for exopolysaccharides with higher antioxidant activities from* Xerocomus badius* cultured in shrimp byproduct medium, Plackett-Burman design, path of steepest ascent, and response surface methodology were explored. Based on the results of Plackett-Burman design and path of steepest ascent, a Box-Behnken design was applied to optimization and the regression models. The optimal cultural condition for high yield and antioxidant activity of the exopolysaccharides was determined to be 10.347% of solid-to-liquid ratio, a 4.322% content of bran powder, and a 1.217% concentration of glacial acetic acid. Culturing with the optimal cultural conditions resulted in an exopolysaccharides yield of 4.588 ± 0.346 g/L and a total antioxidant activity of 2.956 ± 0.105 U/mg. These values are consistent with the values predicted by the corresponding regression models (RSD < 5%).

## 1. Introduction

Free radicals, generated during aerobic respiration, are molecules or molecular fragments containing one or more unpaired electrons and would cause cumulative oxidative damage, resulting in aging and death [1]. Excessive amount of free radicals is called “oxidative stress” [2], which can lead to cell and tissue damage, the dysregulation of redox-sensitive signaling pathways and increases with aging and aging-related degenerative diseases [3]. Antioxidant can safely interact with free radicals, which result in the termination of the chain reaction before vital biological macromolecules are damaged. So it plays an important role to prevent tissue damage from oxidative stress caused by free radicals. It was reported that antioxidants could improve the immune function and decrease the oxidative stress [4, 5]. Consequently, antioxidants, especially natural antioxidants from edible materials, have been increasingly concerned for their reducing of the risk of aging and aging-related degenerative diseases [6, 7]. Thus, numerous natural antioxidants, such as phenolic compounds, organic acid, carotenoids, vitamins, and polysaccharide, have already been utilized in foods, medicines, and cosmetics [8–12].

Polysaccharides derived from fruiting bodies and fermented broths of edible/medicinal mushrooms have been increasingly studied in recent years due to their potential antioxidant properties [10, 12, 13]. These properties have driven research into learning more about production, extraction, and additional bioactivities of polysaccharides [10, 14, 15].

The Boletaceae, a family including several kinds of mushrooms, has been reported to have antioxidants in some of the fruiting bodies, including* Boletus badius*,* Boletus edulis*,* Suillus granulatus*, and* Xerocomus badius*. These antioxidants found in the Boletaceae are mainly phenolic compounds, tocopherols, ascorbic acid, and carotenoids [8]. However, there are few reports describing the antioxidant activities of polysaccharides from the Boletaceae. It was found in our previous study that polysaccharides from liquid fermented* Suillus bovinus* and* B. edulis* cultured in both potato dextrose agar (PDA) and shrimp byproduct medium had antioxidant properties in three assays [16]. But antioxidant activity of exopolysaccharides (EPS) from* X. badius* grown in any medium has not been reported.

Shrimp byproduct, including the head, shell, tail portion, and small fry, is always removed and discarded. However, uncontrolled dumping and hauling of shrimp byproduct had resulted in wasting of a natural resource and eutrophication of the coastal and marine environment [17]. This problem will become increasingly severe without effective measures to change its disposal. Therefore, large-scale recycling of shrimp byproduct has gained a lot of focus. Shrimp byproduct has been reused for fertilizer, feedstuff [18, 19], and recovering nutrients comprising and bioactive substances [20, 21]. However, these methods can only reuse a small portion of the waste resource. Additionally, extraction of bioactive substances requires large quantities of strong acids or bases [22], which may cause secondary pollution of the environment and increases in the cost of disposal. As an alternative recycling method, in this study, the shrimp byproduct was used as the major constituent of medium in liquid fermentation. This method could cut down the costs of production of the EPS from* X. badius* on one hand and result in a practical, environmentally friendly, and high value added way to recycle shrimp byproduct on the other hand.

Therefore, in series, PB design, the steepest ascent method, and RSM were applied to optimize liquid fermentation conditions for optimal production and antioxidant activities of EPS by* X. badius* cultured in shrimp byproducts. The aim of this paper was to provide a theoretical and practical basis for large-scale economical production of EPS with high antioxidant activities from liquid fermented* X. badius*.

## 2. Materials and Methods

### 2.1. Chemicals

Assay kits for screening of total antioxidant (T-AO), antisuperoxide anion radical (ASAR), and antihydroxyl radical (AHR) activities were purchased from Nanjing Jiancheng Bioengineering Research Institute in China. Shrimp byproducts were purchased from a local shrimp processing plant and were pulverized after being dried at 50°C. Potatoes were purchased from a local market. All other chemicals used in this paper were of analytical reagent grade and made in China.

### 2.2. Microorganism and Liquid Fermentation

*X. badius*, maintained in pitched PDA (composed of 200 g/L potato, 20 g/L glucose, and 20 g/L agar powder) medium at 4°C, was activated in Petri dishes in the solid medium (PDA) at 25°C. The strain was cultivated in 250 mL shake-flasks containing 100 mL of liquid medium (PDA, solid medium minus the agar) in a shaking incubator (130 rpm) at 25°C for 3 days, which served as liquid seeds for fermentation. The initial fermentation in liquid medium occurred in 100 mL deionized water containing 1.3% acetic acid and 8.0 g of solid compounds (95% shrimp byproduct and 5% bran powder). The liquid cultivation was carried out in 250 mL shake-flasks containing 100 mL of culture medium, as well as 8 mL of liquid seeds (25°C, 120 rpm). As a control, liquid medium without any fungal species was inoculated in the same conditions.

### 2.3. Cultivation and Conditions Optimization for Antioxidant EPS Production

#### 2.3.1. Plackett-Burman (PB) Design

A total of 8 variables denoted *X*_1–8_ were screened in PB design to determine significant variables that influence the production and antioxidant activity of EPS. Each independent variable was assigned a high (+1) and low (−1) level. In the present study, the low (−1) levels were the same as those in the initial fermentation, whereas the high (+1) levels were 1.25 times the lower levels. Variable *A*, *B*, and *C* served as dummy columns to calculate the standard error. All experimental trials, factors, and their levels are shown in Table 1. The yield and T-AO activity of EPS were the response values. Based on the results obtained by the PB design, the first-order model was

(1) $$\begin{array}{c} Y_{\left(1,2\right)}=\beta_{0}+\sum \beta_{i}X_{i}\;\left(i=1\cdots k\right), \end{array}$$

where *Y*_1_ is the yield and *Y*_2_ is the T-AO activity of EPS, *β*_0_ is a constant, *β*_*i*_ are the regression coefficients, and *X*_*i*_ are the coded independent factors showed in Table 1.

#### 2.3.2. Path of Steepest Ascent

Three key variables were chosen based on the results of the PB design, the center of which was the starting point of the path of steepest ascent. The step-size was determined by the experimenter based on process knowledge and other practical considerations. The direction of steepest ascent (descent) was the direction in which the response increased the most. All factor levels were fixed, except the known key factors identified through the PB design. The path of speed ascent factors and their experimental trials (10 in all) are shown in Table 2.

#### 2.3.3. Response Surface Methodology (RSM)

For the RSM, a Box-Behnken design (BBD) experimental plan with 3 factors at 3 levels and 17 experimental trials in total (Table 3) was used for providing a precise description about relationships between factors and the resulting response. Results of the BBD were fit with a second-order polynomial equation:

(2) $$\begin{array}{c} Y_{\left(1,2\right)}=\beta_{0}+\overset{k}{\underset{i}{\sum }}\beta_{i}X_{i}+\overset{k}{\underset{ii}{\sum }}\beta_{ii}{X_{i}}^{2}+\sum_{i<j}\beta_{ij}X_{i}X_{j}. \end{array}$$

In the equation, *Y*_1_ is the yield of EPS, *Y*_2_ is the T-AO activity of EPS, *β*_0_ is the offset term, *β*_*i*_ are the linear effects, *β*_*ii*_ are the squared terms, *β*_*ij*_ are the interaction effects, and *X*_*i*_ and *X*_*j*_ are the coded independent factors shown in Table 3.

#### 2.3.4. Verification of the Model

The RSM model adequacy was experimentally tested at optimum conditions in order to obtain a maximum yield and antioxidant activities of EPS. Therefore, results obtained in the initial fermentation stage were compared to the predicted values to validate the model.

### 2.4. Yield of Crude EPS Measurement

At the end of cultivation, the fermented products and blank medium were divided into solid constituent and supernatant fluid, respectively, by filtering through gauze. The fermented supernatants were collected, centrifuged at 10,000 rpm for 10 min, filtered through filter paper, and diluted with three times their volume of ethanol (95%). The crude EPS were incubated at 4°C for 12 h and then centrifuged at 8,000 rpm (4°C) for 20 min. The precipitates were collected, washed with 95% ethanol twice, lyophilized, and weighed. Then, 10 mg of the EPS was dissolved with distilled water at 60°C to determine the EPS content by the phenol-sulfuric acid method (where glucose was used as a standard). The yield of EPS was calculated by multiplying the weight of crude EPS by the EPS content. Finally, EPS obtained from the initial and optimal fermentations were dissolved in distilled water for examining their antioxidant activities. For controls, the same treatment was imposed on blank media to gain the crude polysaccharides.

### 2.5. Antioxidant Activities Assay

EPS from* X. badius* cultured in shrimp byproduct medium (before and after optimization), as well as their corresponding blanking medium, were dissolved in distilled water at various concentrations. The T-AO, ASAR, and AHR activities of these samples were measured by using their respective antioxidant assay kits.

According to the specifications described in the kit, the T-AO activity assay was used to measure the ferric-reducing power of the samples. The kit uses phenanthroline substances which change color when antioxidant substances reduce ferrous iron to ferric iron to measure this capacity. The T-AO of vitamin C (20 mM) was measured as a positive control. The absorbance was determined at 520 nm against distilled water by using a UV/vis spectrophotometer (Beijing KWF sci-tech Development Co., Ltd., China). A unit of T-AO activity is defined as the amount of 1 mg of a certain sample that increased the absorbance of the reaction system by 0.01 at 37°C for 1 min.

The assay of ASAR activity is based on the concept that some antioxidant substances inhibit the generation of superoxide anions in reactions containing xanthine and xanthine oxidase. This inhibition lowers the absorbance after an electron transfer substance and chromogenic agent are added. The absorbance was measured at 550 nm against distilled water using a UV/vis spectrophotometer. A unit of ASAR activity is defined as the amount the superoxide anion inhibited by 1 g of a certain sample, which equaled the effect 1 mg of vitamin C had, at 37°C for 40 min.

The assay of AHR activity is based on the concept that in a Fenton reaction some antioxidant substances have inhibitive power on the production of hydroxyl radicals. This inhibitive process then reduces the absorbance of the reaction system after an electron acceptor and chromogenic agent are added. The AHR activity of vitamin C (5 mM) was used again as a positive control. The absorbance was measured at 550 nm against distilled water using a UV/vis spectrophotometer. A unit of AHR is defined as the amount of 1 mg of sample that reduces H_2_O_2_ by 1 mM at 37°C for 1 min.

The T-AO, ASAR, and AHR activities were formulated according to the following equations:

(3) T-AO activity (U/mg)=ODE−ODC×N0.01×30×C,ASAR activity (U/g)=ODC−ODE×CS×1000 mLODC−ODS×C,AHR activity (U/mg)=ODC−ODE×8.824ODC−ODB×C×V,

where OD_E_ is the optical density of the experimental group and OD_C_ is the control group, *C* is the concentration of samples (mg/mL), *N* is the dilution of the reaction system, OD_S_ is the standard group, *C*_S_ is the concentration of vitamin C (used as a standard, 0.15 mg/mL), OD_B_ is the blank sample, 8.824 is the molar concentration of H_2_O_2_ (0.03%), and *V* is the volume of the samples.

### 2.6. Statistical Analysis

All experiments in the present study were carried out in triplicate. Results of initial fermentation stage, the steepest ascent, and verification experiments were statistically analyzed by analysis of variance (ANOVA) and *t*-test with SPSS 16.0 software. The PB and RSM experimental design were done with the Design-Expert 7.1.6 software.

## 3. Results and Discussion

### 3.1. Bioconversion Medium and Conditions Optimization for EPS Production

#### 3.1.1. PB Design for Screening of Bioconversion Conditions

Of the 8 total bioconversion conditions screened using PB statistical experimental design, the yield and T-AO activity of crude EPS were chosen as the response variables. The yield of EPS from* X. badius* grown in liquid shrimp byproduct medium ranged from 0.091 ± 0.010 to 3.834 ± 0.124 g/L and their T-AO activity ranged from 0.345 ± 0.022 to 2.217 ± 0.112 U/mg. The first-order models of the yield and T-AO activity of EPS in terms of coded factors were established based on these results, which are as follows:

(4) Y1=1.700+0.328X1−0.413X2−0.034X3+0.101X4−0.044X5−0.585X6+0.305X7+0.069X8,Y2=1.382+0.258X1−0.277X2+0.154X3+0.138X4−0.026X5−0.343X6+0.035X7−0.139X8,

where *Y*_1_ is the yield of EPS, *Y*_2_ is the T-AO activity of the EPS, and *X*_1–8_ are the coded factors shown in Table 1.

**Figure 1:**
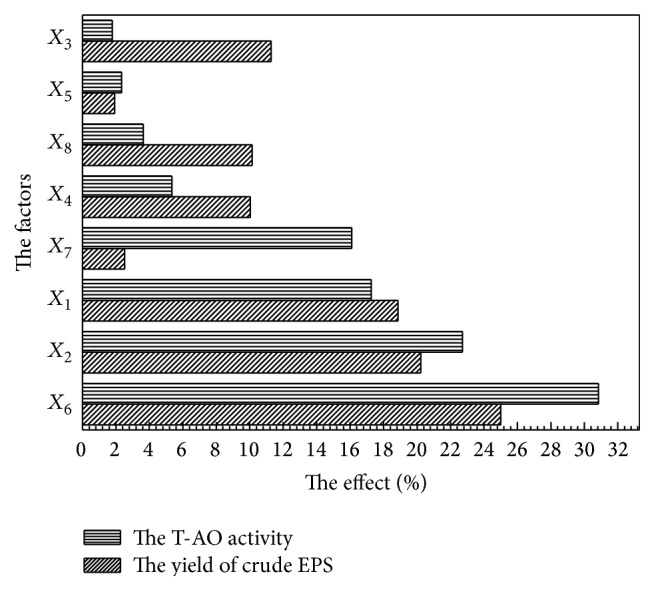
Pareto plot for Plackett-Burman parameter estimates for 8 factors.

The results of regression and ANOVA analysis of PB design for the yield of EPS (Table 4) showed that 5 of the 8 factors had a significant effect on the yield of crude EPS (*P* < 0.05). Furthermore, the significance of the factors affecting the yield was demonstrated by the Pareto plot (Figure 1), which was *X*_6_ > *X*_2_ > *X*_1_ > *X*_7_ > *X*_4_ > *X*_8_ > *X*_5_ > *X*_3_. In ANOVA analysis of PB design, the determination coefficient (*R* ^2^) was the value for checking the goodness of fit of the regression model. The *R* ^2^ value of the yield of EPS (0.9960) meant that 99.60% of the variability of the response was captured by the model. The Model *F*-Value of 92.357 implied the model was significant (*P* = 0.0017) and there was only a 0.40% chance that a “Model *F*-Value” this large could occur due to noise. The Pred *R* ^2^ of 0.9353 was in reasonable agreement with the Adj *R* ^2^ of 0.9852. The Adeq Precision value measures the signal-to-noise ratio. A ratio greater than 4 is desirable. The ratio of 36.024 indicated an adequate signal. This model, therefore, can be used to navigate the design space.

The results of regression and ANOVA analysis of PB design for the T-AO activity of crude EPS (Table 4) implied that 6 of the 8 factors had a significant effect on the T-AO activity of crude EPS (*P* < 0.05). Furthermore, the significance of the factors affecting the T-AO activity was demonstrated by the Pareto plot (Figure 1), which was *X*_6_ > *X*_2_ > *X*_1_ > *X*_3_ > *X*_8_ > *X*_4_ > *X*_7_ > *X*_5_. The *R* ^2^ value of the regression model of the T-AO activity of crude EPS (0.9895) meant that 98.95% of the variability of the response was captured by the model. The Model *F*-value of 35.366 implied the model was significant (*P* = 0.0069) and there was only a 1.05% chance that a “Model *F*-Value” this large could occur due to noise. The Pred *R* ^2^ of 0.8321 was in reasonable agreement with the Adj *R* ^2^ of 0.9615. The Adeq Precision of 17.266 (far greater than 4) indicated an adequate signal-to-noise ratio. Therefore, this model could be used to navigate the design space.

In some reports, factors with confidence levels above 80% [23] or 85% [24] were chosen as major factors for further optimization. In other studies, 3 to 5 significant factors were chosen [25]. In this paper, the top 3 significant variables (*X*_6_, concentration of glacial acetic acid; *X*_1_, solid-to-liquid ratio; *X*_2_, content of bran powder) were considered the major factors. Other variables were excluded from the following optimization due to significantly smaller contributions to the models. The factors of *X*_4_ (inoculation amount of mycelium) and *X*_7_ (cultural temperature) were used in all trials at their high (+1) level due to positive regression coefficients in the two models, whereas *X*_5_ (rotation rate) was used at a low (−1) level due to a negative regression coefficient in the two models. Regression coefficients of *X*_3_ (volume of liquid medium) varied depending on the model and were negative in the model of the yield and positive in the model of the T-AO activity, which is contrary to the regression coefficients of *X*_8_ (cultivation time). The level of these two factors was defined according to regression coefficients in the model of the T-AO activity, which were larger. Based on first-order models, the path of steepest ascent was done to find the proper region of maximum yields and T-AO activity of crude EPS. The path of steepest ascent started from the center of the factorial design and moved along the path in which the solid-to-liquid ratio was increasing, while the content of bran powder and concentration of glacial acetic acid were decreasing.

#### 3.1.2. Path of Steepest Ascent for Locating the Region of Optimum Response

The results of the path of steepest ascent experiments (Table 2) indicated that the optimum neighborhood of the yields of crude EPS was 4.346 ± 0.124 g/L when the solid-to-liquid ratio was 8.70%, the content of bran powder was 4.55%, and the concentration of glacial acetic acid was 1.16%. The optimum neighborhood of the T-AO activity was 2.787 ± 0.171 U/mg when the solid-to-liquid ratio was 9.10%, the content of bran powder was 4.20%, and the concentration of glacial acetic acid was 1.06%. The optimal levels of the three factors in BBD are defined in Table 3 when both the yield and T-AO activity are taken into consideration.

#### 3.1.3. Box-Behnken Design (BBD) for Further Optimization

Three variables (*X*_1_, *X*_2_, and *X*_6_) were used to determine the optimum values by response surface methodology via BBD. The levels of the three factors for the BBD experiments were fixed according to the results of the path of steepest ascent methods. The design matrix and the corresponding responses of BBD are shown in Table 3. It was indicated that variation in the yields and T-AO activity of crude EPS depended upon the culture conditions considered.

The results of the BBD were fit with the following second-order model in terms of actual factors:

(5) Y1=−187.694+28.098X1+12.588X2+17.703X6+0.062X1X2+0.028X1X6−1.680X2X6−1.377X12−1.729X22−4.032X62,Y2=−125.945+14.386X1+9.906X2+54.742X6−0.110X1X2−0.651X1X6−2.456X2X6−0.637X12−0.682X22−15.468X62,

where *Y*_1_ is the yield of EPS, *Y*_2_ is the T-AO activity of the EPS, and *X*_1_, *X*_2_, and *X*_6_ are the coded factors shown in Table 1.

The relationships between the dependent and independent variables are shown in (5). The coefficients with one factor, two factors and those with second-order terms represented the effect of a particular factor, the interaction between the two factors, and the quadratic effect, respectively. According to the results of the regression and ANOVA analyses on the models of the BBD (Table 5), the Model *F*-Value of 364.998 and 19.041 implied the models were significant (*P* < 0.0001 and *P* = 0.0004). The *R* ^2^ values for the two regression models were 0.9979 and 0.9608, respectively, which meant that 99.79% of the variability of the yield and 96.08% of the T-AO activity were captured by the models. The Pred *R* ^2^ for the two regression models of 0.9786 and 0.8002 were in reasonable agreement with their Adj *R* ^2^ of 0.9951 and 0.9103, respectively, indicating a high degree of correlation between the experimental and predicted values. The Adeq Precision values of 59.423 and 10.885 (greater than 4) indicate there is an adequate signal-to-noise ratio. The *P* values of the lack of fit for the two regression models (0.2688 and 0.7422) were higher than 0.05, making them not significant. These results implied that the models describe variability of the yield and T-AO activity affected by the factors successfully.

**Figure 2:**
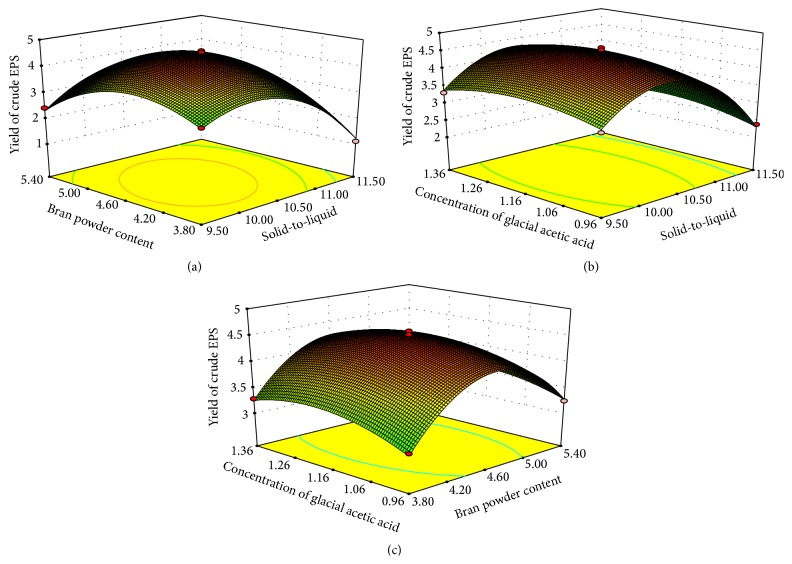
Response surface plots of the yield of crude EPS. (a) Interaction of the solid-to-liquid ratio and bran powder content; (b) interaction of the solid-to-liquid ratio and the concentration of glacial acetic acid; (c) interaction of the bran powder content and the concentration of glacial acetic acid.

**Figure 3:**
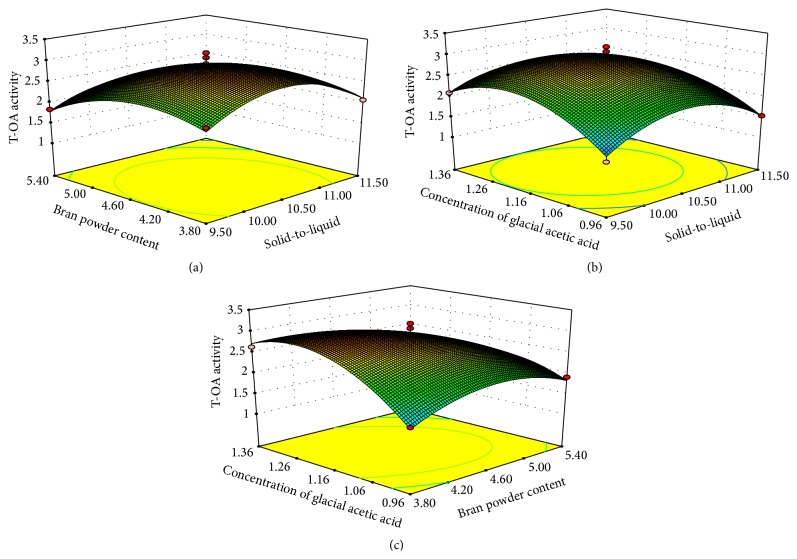
Response surface plots of the T-OA activity of crude EPS. (a) Interaction of the solid-to-liquid ratio and the bran powder content; (b) interaction of the solid-to-liquid ratio and the concentration of glacial acetic acid; (c) interaction of the bran powder content and the concentration of glacial acetic acid.

The three-dimensional response surface plots were constructed with Design-Expert 7.1.6 software to estimate the effects of the factors and their interactions on the yield (Figures 2(a)–2(c)) and T-AO activity (Figures 3(a)–3(c)) of crude EPS. As shown in Figures 2(a)–2(c), the yield of crude EPS rose as the solid-to-liquid ratio increased from 9.50% to 10.315% and declined when the solid-to-liquid ratio ranging was beyond 10.315%. The effect of bran powder content and concentration of glacial acetic acid on yield of crude EPS also varied within the tested range. The optimal condition predicted for the highest yield of crude EPS from* X. badius* grown in liquid shrimp byproduct medium was when the solid-to-liquid ratio was 10.315%, the content of bran powder was 4.364%, and the concentration of glacial acetic acid was 1.269%. As a result, the highest yield of crude EPS was predicted as 4.649 g/L. Figures 3(a)–3(c) illustrate the interaction between the three factors corresponding to the T-AO activity of crude EPS. The elliptical contours indicate that the T-AO activity increases when the solid-to-liquid ratio increases up to 10.00–10.50% but decreases rapidly beyond this. The effect of bran powder content and concentration of glacial acetic acid on the T-AO activity also varied within the tested range. The optimum cultural condition for the highest T-AO activity of crude EPS from* X. badius* grown in liquid shrimp byproduct medium was predicted to be when the solid-to-liquid ratio was 10.391%, the bran powder content was 4.238%, and the concentration of glacial acetic acid was 1.214%. As a result, T-AO activity was predicted as 3.028 U/mg.

Finally, the model predicted that the optimal cultural conditions for both increased yield and T-AO activity of crude EPS are when the solid-to-liquid ratio is 10.347%, the bran powder content is 4.322%, and the concentration of glacial acetic acid is 1.217%. Under the optimal conditions, the yield and T-AO activity of crude EPS were predicted as 4.629 g/L and 3.027 U/mg.

#### 3.1.4. Validation Experiments

Experiments were carried out in triplicate using the optimum cultural conditions predicted for the highest yield of crude EPS, the highest T-AO activity of crude EPS, and the highest combined yield and T-AO activity of crude EPS obtained from BBD in order to validate the suitability of the regression models for predicting optimum response values. Using the parameters for the optimal cultural condition for the highest yield of crude EPS, the measured yield was determined to be 4.668 ± 0.213 g/L, which is not significantly different from the predicted yield of 4.649 g/L (*P* > 0.05). Using the parameters for the optimal cultural conditions for the highest T-AO activity, the measured T-AO activity was determined to be 2.979 ± 0.131 U/mg, which is not significantly different from the predicted biomass of 3.032 U/mg (*P* > 0.05). Finally, using the parameters for the optimal cultural conditions for both the highest yield and greatest T-AO activity of crude EPS, the measured yield and T-AO activity of crude EPS were 4.588 ± 0.346 g/L and 2.956 ± 0.105 U/mg, respectively. Again these values are not significantly different from the predicted values of the yield of crude EPS 4.629 g/L and the T-AO activity of 3.027 U/mg, respectively (*P* > 0.05 for both). The optimum models improved the average yield of crude EPS by 123.456% compared to the yield obtained before optimization (2.089 ± 0.114 g/L). Additionally, T-AO activity of crude EPS improved by 61.814% under optimal cultural conditions for the highest T-AO activity compared with the T-AO activity obtained before optimization (1.841 ± 0.071 U/mg). Furthermore, optimizing for both yield and T-AO activity of crude EPS improved the yield and activity by 119.627% and 60.565%, respectively. The results imply that the optimized conditions were suitable for high yield and T-AO activity of crude EPS from* X. badius* grown in liquid shrimp byproduct medium.

The combination of Plackett-Burman, path of steepest ascent, and Box-Behnken design was effective and reliable in selecting the statistically significant factors and optimal levels of those factors for the highest yield and T-AO activity of crude EPS in this study. The shrimp byproduct medium contains large amounts of nutritive components [26] and has been fermented by lactic acid bacteria to recover chitosan and chitin [27, 28]. However, the shrimp byproduct is subalkaline [27]; therefore, glacial acetic acid (about 1%) was needed to begin fermentation. In the present study, 1.25% of glacial acetic acid was added to the medium for the highest yield and T-AO activity when the solid-to-liquid ratio was 10.30%. The addition of acetic acid resulted in a pH value of 5.294 ± 0.085. Initial medium pH may affect metabolite formation and product biosynthesis of cells, due to its effect on the uptake of various nutrients. It is reported that EPS production in cultures of* Sclerotium glucanicum* and* Ganoderma lucidum* was greatly affected by culture pH, and the optimum pH was 3.5–4.5 over long-term cultivation [29]. The optimum pH value for high yield and T-AO activity of crude EPS was 5.294 for* X. badius* cultured in shrimp byproduct medium in the present study. This value is higher than that of* S. glucanicum* and* G. lucidum*.

### 3.2. T-AO, ASAR Activity, and AHR Activity of the EPS

In the present study, in addition to the T-AO activity, the antioxidant activities of EPS from* X. badius* cultured in shrimp byproduct medium were evaluated with ASAR and AHR activity assays.

The T-AO activity of the polysaccharide from its corresponding blank medium was determined to be 0.079 ± 0.017 U/mg, which was far lower than the T-AO activity of EPS gained before and after optimization (*P* < 0.001). The T-AO activity of vitamin C (25 mM, 3.369 ± 0.227 U/mg) was measured simultaneously, which was 1.140 times greater than the T-AO activity of the EPS gained under optimum conditions. ASAR activity of the EPS gained after optimization of 144.718 ± 2.439 U/g indicated that 1 g of the EPS could inhibit the same amount of the superoxide anion as 144.718 mg of vitamin C did. This value was higher than that of the polysaccharide value obtained from the blank medium (12.397 ± 0.874 U/g, *P* < 0.0001) and improved by 143.039% compared to that of EPS gained before optimization (59.545 ± 1.331 U/g). The AHR activity of the EPS was determined to be 59.714 ± 1.952 U/mg, which was higher than the value obtained from the blank medium (11.641 ± 0.445 U/mg, *P* < 0.001), and improved by 11.323% compared to that of EPS gained before optimization (53.642 ± 1.041 U/mg). The AHR activity of the EPS gained under the optimum conditions was 86.026% of the AHR activity of vitamin C (5 mM, 69.414 ± 2.016 U/mg).

Results of the present study demonstrate that antioxidant activities of EPS from* X. badius* are far higher than their corresponding blanking medium. This indicates that there is a higher antioxidant activity produced after treatment with the shrimp byproduct by* X. badius*. Furthermore, AHR and ASAR activity were significantly improved after optimization, indicating that using the T-AO activity as a response variable for the highest antioxidant activities was a practical and suitable solution.

T-AO, AHR, and ASAR activity of crude EPS cultured in several different kinds of fungal medium were previously investigated. Yan et al. [30] determined that the T-AO and ASAR capacity of crude EPS from the fungus* Hypsizygus marmoreus* varies depending on the type of fermentation media. The highest T-AO capacity of EPS from* H. marmoreus* was 0.813 U/mg. Xie et al. [31] reported that the T-AO capacity of EPS from* H*.* marmoreus* grown in kelp waste was 1.18 U/mg. Gao et al. [16] reported that the T-AO, ASAR, and AHR activity of crude EPS from* B. edulis* cultured in shrimp byproduct was 1.718 U/mg, 46.703 U/g, and 48.255 U/mg and* S. bovinus* was determined to be 1.053 U/mg, 25.797 U/g, and 52.149 U/mg. In this study, after the cultural conditions were optimized, the EPS from* X. badius* had a T-AO activity of 2.596 U/mg, ASAR activity of 144.718 U/g, and AHR activity of 59.714 U/mg, which are far greater than the previous reports of antioxidant activity from fungus.

## 4. Conclusions

The main factors that affect the yield and antioxidant activities of crude EPS from* X. badius* and their optimum level were screened for the first time. The solid-to-liquid ratio, bran powder content, and concentration of glacial acetic acid were determined to be the main factors. The optimum level of these factors was determined to be 10.347%, 4.322%, and 1.217%, respectively. Under the optimum conditions, yield of the crude EPS reached 4.588 g/L and improved by 123.456% compared to the value determined before optimization. The T-AO activity of the crude EPS reached 2.596 g/L and improved by 61.814% compared to the value before optimization. Furthermore, the AHR activity and ASAR activity were significantly improved after optimization. The study is the foundation of a low-cost industrial scale production of antioxidant polysaccharides.

## Figures and Tables

**Table 1:** Plackett-Burman experimental design matrix for screening the cultural medium and conditions with mycelium biomass and yield of crude EPS as response.

Runs	Experimental factors											Yields of EPS (g/L)	T-AO activity (U/mg)
	*X*_1_	*X*_2_	*A*	*X*_3_	*X*_4_	*B*	*X*_5_	*X*_6_	*C*	*X*_7_	*X*_8_		
1	1 (10.0)	−1 (5.0)	1	−1 (100)	1 (10.0)	1	−1 (120)	−1 (1.30)	−1	1 (30)	1 (96)	3.634 ± 0.124	2.146 ± 0.130
2	1 (10.0)	−1 (5.0)	1	−1 (100)	−1 (8.0)	−1	1 (150)	1 (1.63)	1	1 (30)	−1 (72)	1.956 ± 0.171	1.371 ± 0.098
3	−1 (8.0)	1 (6.5)	−1	−1 (100)	−1 (8.0)	1	1 (150)	−1 (1.30)	1	1 (30)	1 (96)	1.724 ± 0.163	0.864 ± 0.074
4	−1 (8.0)	−1 (5.0)	1	1 (125)	1 (10.0)	−1	1 (150)	−1 (1.30)	1	−1 (25)	1 (96)	2.135 ± 0.174	1.776 ± 0.106
5	1 (10.0)	−1 (5.0)	−1	1 (125)	−1 (8.0)	1	−1 (120)	1 (1.63)	1	−1 (25)	1 (96)	1.481 ± 0.133	1.542 ± 0.095
6	−1 (8.0)	−1 (5.0)	−1	1 (125)	1 (10.0)	1	1 (150)	1 (1.63)	−1	1 (30)	−1 (72)	1.519 ± 0.147	1.557 ± 0.107
7	1 (10.0)	1 (6.5)	−1	−1 (100)	1 (10.0)	−1	1 (150)	1 (1.63)	−1	−1 (25)	1 (96)	0.935 ± 0.131	0.785 ± 0.034
8	1 (10.0)	1 (6.5)	−1	1 (125)	1 (10.0)	−1	−1 (120)	−1 (1.3)	1	1 (30)	−1 (72)	2.493 ± 0.195	2.217 ± 0.112
9	−1 (8.0)	−1 (5.0)	−1	−1 (100)	−1 (8.0)	−1	−1 (120)	−1 (1.30)	−1	−1 (25)	−1 (72)	2.061 ± 0.144	1.563 ± 0.083
10	1 (10.0)	1 (6.5)	1	1 (125)	−1 (8.0)	1	1 (150)	−1 (1.30)	−1	−1 (25)	−1 (72)	1.664 ± 0.209	1.782 ± 0.091
11	−1 (8.0)	1 (6.5)	1	−1 (100)	1 (10.0)	1	−1 (120)	1 (1.63)	1	−1 (25)	−1 (72)	0.091 ± 0.010	0.637 ± 0.047
12	−1 (8.0)	1 (6.5)	1	1 (125)	−1 (8.0)	−1	−1 (120)	1 (1.63)	−1	1 (30)	1 (96)	0.704 ± 0.017	0.345 ± 0.022

Notes: *X*_1_, solid-to-liquid ratio (%, m/v); *X*_2_, bran powder content (%); *X*_3_, volume of liquid medium (mL); *X*_4_, inoculation amount of mycelium (%); *X*_5_, rotation rate (rpm); *X*_6_, concentration of glacial acetic acid (%); *X*_7_, cultural temperature (°C); *X*_8_, cultivation time (h); *A*, *B*, and *C* refer to dummy columns.

**Table 2:** Experimental trials in the path of steepest ascent (descent).

Trials	Experimental factors			Yields of EPS (g/L)	T-AO activity (U/mg)
	*X*_1_	*X*_2_	*X*_6_		
1	9.00	5.80	1.46	0.19 ± 0.022	0.337 ± 0.027
2	9.50	5.40	1.36	1.418 ± 0.015	1.365 ± 0.102
3	10.00	5.00	1.26	3.548 ± 0.269	2.154 ± 0.114
4	10.50	4.60	1.16	4.346 ± 0.124	2.779 ± 0.138
5	11.00	4.20	1.06	3.351 ± 0.169	2.787 ± 0.171
6	11.50	3.80	0.96	3.015 ± 0.184	1.228 ± 0.103
7	12.00	3.40	0.86	1.644 ± 0.093	1.162 ± 0.087
8	12.50	3.00	0.76	0.136 ± 0.055	0.649 ± 0.046

**Table 3:** Box-Behnken designs of different factors with their responses.

Trials	Experimental factors			Yields of EPS (g/L)	T-AO activity (U/mg)
	*X*_1_	*X*_2_	*X*_6_		
1	0 (10.5)	1 (5.4)	1 (1.36)	2.547 ± 0.077	1.296 ± 0.326
2	1 (11.5)	−1 (3.8)	0 (1.16)	1.909 ± 0.108	2.068 ± 0.083
3	0 (10.5)	0 (4.6)	0 (1.16)	4.412 ± 0.173	3.072 ± 0.860
4	0 (10.5)	−1 (3.8)	1 (1.36)	4.088 ± 0.156	2.633 ± 0.122
5	0 (10.5)	0 (4.6)	0 (1.16)	4.522 ± 0.151	2.929 ± 0.096
6	0 (10.5)	−1 (3.8)	−1 (0.96)	3.366 ± 0.231	1.674 ± 0.078
7	−1 (9.5)	0 (4.6)	1 (1.36)	3.515 ± 0.095	2.092 ± 0.113
8	0 (10.5)	0 (4.6)	0 (1.16)	4.483 ± 0.07	3.184 ± 0.105
9	1 (11.5)	0 (4.6)	1 (1.36)	2.634 ± 0.229	1.676 ± 0.098
10	1 (11.5)	0 (4.6)	−1 (0.96)	2.384 ± 0.072	1.534 ± 0.128
11	1 (11.5)	1 (5.4)	0 (1.16)	1.023 ± 0.094	1.251 ± 0.086
12	0 (10.5)	0 (4.6)	0 (1.16)	4.588 ± 0.186	2.879 ± 0.104
13	0 (10.5)	0 (4.6)	0 (1.16)	4.496 ± 0.108	2.602 ± 0.094
14	−1 (9.5)	1 (5.4)	0 (1.16)	2.025 ± 0.104	1.838 ± 0.083
15	−1 (9.5)	0 (4.6)	−1 (0.96)	3.288 ± 0.144	1.429 ± 0.114
16	0 (10.5)	1 (5.4)	−1 (0.96)	2.900 ± 0.117	1.909 ± 0.077
17	−1 (9.5)	−1 (3.8)	0 (1.16)	3.109 ± 0.139	2.303 ± 0.080

**Table 4:** ANOVA analysis for the Plackett-Burman factorial model of yield and T-AO activity of crude EPS.

Source	Yield of crude EPS (g/L)					T-AO activity (U/mg)				
	Sum of squares	df	Mean square	*F*-Value	*P* value	Sum of squares	df	Mean square	*F*-Value	*P* value
Model	8.966	8	1.121	92.357	0.0017	3.899	8	0.487	35.366	0.0069
*X*_1_	4.111	1	4.111	338.816	0.0003	1.408	1	1.408	102.194	0.0021
*X*_2_	0.014	1	0.014	1.132	0.3654	0.286	1	0.286	20.763	0.0198
*X*_3_	0.123	1	0.123	10.155	0.0498	0.227	1	0.227	16.483	0.0269
*X*_4_	2.231	1	2.231	183.843	0.0009	0.921	1	0.921	66.852	0.0038
*X*_5_	0.057	1	0.057	4.708	0.1185	0.232	1	0.232	16.844	0.0262
*X*_6_	0.024	1	0.024	1.944	0.2576	0.008	1	0.008	0.600	0.4950
*X*_7_	1.287	1	1.287	106.067	0.0020	0.801	1	0.801	58.148	0.0047
*X*_8_	1.119	1	1.119	92.195	0.0024	0.014	1	0.014	1.041	0.3826
Residual	0.036	3	0.012			0.041	3	0.014		
Cor total	9.002	11				3.940	11			

	*R* ^2^ = 0.9960; Adj *R* ^2^ = 0.9852; Pred *R* ^2^ = 0.9353; Adeq Precision = 36.024					*R* ^2^ = 0.9895; Adj *R* ^2^ = 0.9615; Pred *R* ^2^ = 0.8321; Adeq Precision = 17.266				

**Table 5:** ANOVA analysis for quadratic regression model of the yield and T-AO activity of crude EPS in BBD.

Source	Yield of crude EPS (g/L)					T-AOA (U/mg)				
	Sum of squares	df	Mean square	*F*-Value	*P* value	Sum of squares	df	Mean square	*F*-Value	*P* value
Model	18.603	9	2.067	364.998	<0.0001	6.312	9	0.701	19.041	0.0004
*X*_1_	1.987	1	1.987	350.869	<0.0001	0.160	1	0.160	4.356	0.0753
*X*_2_	0.089	1	0.089	15.798	0.0054	0.166	1	0.166	4.496	0.0717
*X*_6_	1.977	1	1.977	349.111	<0.0001	0.710	1	0.710	19.287	0.0032
*X*_1_ *X*_2_	0.000	1	0.000	0.023	0.8829	0.068	1	0.068	1.842	0.2168
*X*_1_ *X*_6_	0.010	1	0.010	1.731	0.2298	0.031	1	0.031	0.841	0.3896
*X*_2_ *X*_6_	0.289	1	0.289	51.015	0.0002	0.618	1	0.618	16.772	0.0046
*X*_1_^2^	7.982	1	7.982	1409.456	<0.0001	1.680	1	1.680	45.618	0.0003
*X*_2_^2^	0.119	1	0.119	21.009	0.0025	1.612	1	1.612	43.760	0.0003
*X*_6_^2^	5.158	1	5.158	910.869	<0.0001	0.802	1	0.802	21.778	0.0023
Residual	0.040	7	0.006			0.258	7	0.037		
Lack of fit	0.023	3	0.008	1.914	0.2688	0.063	3	0.021	0.431	0.7422
Pure error	0.016	4	0.004			0.195	4	0.049		
Cor total	18.643	16				6.570	16			

	*R* ^2^ = 0.9979; Adj *R* ^2^ = 0.9951; Pred *R* ^2^ = 0.9786; Adeq Precision = 59.423					*R* ^2^ = 0.9608; Adj *R* ^2^ = 0.9103; Pred *R* ^2^ = 0.8002; Adeq Precision = 10.8855				
